# Putative DHHC-Cysteine-Rich Domain S-Acyltransferase in Plants

**DOI:** 10.1371/journal.pone.0075985

**Published:** 2013-10-14

**Authors:** Xiaowei Yuan, Shizhong Zhang, Meihong Sun, Shiyang Liu, Baoxiu Qi, Xinzheng Li

**Affiliations:** 1 State Key Laboratory of Crop Biology, Shandong Agricultural University, Tai-An, Shandong 271018, China; 2 National Research Center for Apple Engineering and Technology, Shandong Agricultural University, Tai-An, Shandong 271018, China; 3 Huasheng Agriculture Limited Liability Company, Qingzhou, Shandong 262500, China; 4 Qingzhou City Bureau of Agriculture, Qingzhou, Shandong 262500, China; Wuhan University, China

## Abstract

Protein S-acyltransferases (PATs) containing Asp-His-His-Cys within a Cys-rich domain (DHHC-CRD) are polytopic transmembrane proteins that are found in eukaryotic cells and mediate the S-acylation of target proteins. S-acylation is an important secondary and reversible modification that regulates the membrane association, trafficking and function of target proteins. However, little is known about the characteristics of PATs in plants. Here, we identified 804 PATs from 31 species with complete genomes. The analysis of the phylogenetic relationships suggested that all of the PATs fell into 8 groups. In addition, we analysed the phylogeny, genomic organization, chromosome localisation and expression pattern of PATs in *Arabidopsis*, *Oryza sative*, *Zea mays* and *Glycine max*. The microarray data revealed that *PATs* genes were expressed in different tissues and during different life stages. The preferential expression of the *ZmPATs* in specific tissues and the response of *Zea mays* to treatments with phytohormones and abiotic stress demonstrated that the PATs play roles in plant growth and development as well as in stress responses. Our data provide a useful reference for the identification and functional analysis of the members of this protein family.

## Introduction

S-acylation refers to the reversible post-translational attachment of an acyl group to a cysteine residue via a thioester linkage [1], [2]. The most common fatty acid attached to cysteines is palmitate, and S-acylation is often called S-palmitoylation. Other acyl groups with different chain lengths and degrees of unsaturation can also be added to cysteines in a similar manner [3], [4]. S-acylation is one of a group of lipid modifications that occur on eukaryotic proteins [1]. S-acyl modification mainly affects membrane attachment and trafficking of proteins [5], [6]. S-acyl modification is required for the dynamic association of proteins with membrane subdomains and for the cycling between different cellular membranes [7]–[9]. In addition, S-acylation can influence the stability of proteins, modulate the functions of proteins and mediate interactions between different proteins [9], [10].

S-acyl modification of proteins is carried out by S-palmitoyltransferases, referred to as protein acyltransferase (PATs) [1]. Protein acylation was first described over 30 years ago [11], [12], and in the following years, several hundred acylated proteins were identified. However, the identification of PATs was a more recent discovery. The major breakthrough in this area came from seminal work performed in the yeast *S. cerevisiae*. In 2002, two independent groups identified Akr1 and Erf2 as PATs for the casein kinase Yck2 and the small GTPase Ras2 [13]–[15], respectively. Sequence analyses of the yeast PATs revealed that both Akr1 and Erf2 contain a conserved Asp-His-His-Cys within a Cys-Rich Domain (DHHC-CRD) [16], [17]. This domain was first described in a novel human pancreatic cDNA library clone and a *Drosophila* open reading frame called DNZ1 [17], [18]. The DHHC-CRD is a 51-amino acid domain and is a variant of the C2H2 zinc finger motif [17]. In addition, mutational analyses revealed that the Cys residue of the DHHC stretch is necessary for auto-acylation and for the modification of target proteins [14], [19]. Therefore, this domain appears to represent the active site of these enzymes.

So far, many proteins containing the DHHC-CRD domain have been identified in eukaryotes, including 7 in yeast, 24 in mice, 23 in humans and 24 in *Arabidopsis* [20], [21]. Among these DHHC-CRD proteins, 6 in yeast (Akr1, Erf2, Swf1, Pfa3, Pfa4 and Pfa5) [13], [15], [20], [22]–[24], 16 in mammals (DHHC2–9, 12, 15, 17–21 and 22) and 2 in *Arabidopsis* have already been confirmed to be PATs [20], [25], [26]. Therefore, it is currently thought that all DHHC-domain-containing proteins may function as S-acyltransferases.

In plants, only two PATs have been characterised in detail, TIP1 and PAT10. A screen of *Arabidopsis* mutants resulted in the identification of TIP GROWTH DEFECTIVE1 (TIP1) (At5g20350), which displays a pleiotropic phenotype with defects in cell expansion, root hair and pollen tube growth and an overall dwarf phenotype [27], [28]. Mapping of the mutant allele revealed that TIP1 encodes a DHHC-CRD-containing protein, and it was shown that this protein is indeed S-acylated [25]. Moreover, TIP1 contains N-terminal ankyrin repeats, related to the yeast PAT Akr1, and it is able to complement the PATs function in the *akr1Δ* yeast strain, confirming that TIP1 has a PAT function [25]. The other PAT that has been well characterised is PAT10, which is critical for development and salt tolerance in *Arabidopsis thaliana* [26]. PAT10 regulates the tonoplast localisation of several calcineurin B–like proteins (CBLs), including CBL2, CBL3 and CBL6, whose membrane association also depends on palmitoylation [26]. Loss of *PAT10* function resulted in pleiotropic growth defects, including smaller leaves, dwarfism and sterility [26]. In addition, the protein localisations of 24 *Arabidopsis* PATs were examined last year. *Arabidopsis* PATs proteins display a complex targeting pattern, and they have been detected in the endoplasmic reticulum, Golgi, endosomes and at the vacuolar membrane [7].

The objective of this study was to identify the complete set of putative S-acyltransferases in plants which the genome sequence is available. With the development of comparative genomics, it is now possible to analyse proteins from the same protein family among different species. Recent draft genome sequences for plants offer the opportunity to investigate the *PATs* genes of plants using genomes that have only recently been completely sequenced. We first identified 804 putative PAT proteins in 31 species and then analysed the phylogenetic relationships of these proteins. We analysed the structure, chromosome location and expression patterns of *PAT* genes in *Arabidopsis*, *Oryza sative*, *Zea mays* and *Glycine max* using bioinformatics data and surveyed the expression patterns of the *PATs* genes in *Zea mays* as well as their responses to four phytohormones (6-BA, IAA, SA and ABA) and three abiotic stress mimic treatments (NaCl, PEG and mannitol) using real-time PCR. The results showed that PATs may play important roles in plants growth and development. This study provides a foundation for the cloning and further functional analysis of each member of this protein family.

## Materials and Methods

### Identification of PAT genes in plants

To identify members of the PAT gene family in plants, we collected the known PAT genes in Arabidopsis and analysed the domains of the PAT peptide sequences using the PFAM search tool [29] and the SMART tool [30]. Two different approaches were then performed. First, all of the known Arabidopsis PAT gene sequences were used as query sequences to perform multiple database searches against proteome and genome files downloaded from the Phytozome database (www.phytozome.net/) [31] and PlantGDB database (www.plantgdb.org/) [32]. Stand-alone versions of BLASTP [33] and TBLASTN (http://blast.ncbi.nlm.nih.gov), which are available from NCBI, were used with an e-value cut-off of 1e-003 [34]. All protein sequences derived from the collected candidate PAT genes were examined using the domain analysis programs PFAM (http://pfam.sanger.ac.uk/) and SMART (http://smart.embl-heidelberg.de/) with the default cut-off parameters. Second, we analysed the domains of all plant peptide sequences using a Hidden Markov Model (HMM) analysis with PFAM searching [35]. We then obtained the sequences with the PF01529 PFAM number that contained a typical DHHC RING domain from the plant genome sequences using a Perl-based script. Finally, all protein sequences were compared with known PAT proteins using ClustalX (http://www.clustal.org/) to verify the sequences that were candidate PAT proteins [36]. The isoelectric points and molecular weights of the PAT proteins were obtained with the help of proteomics and sequence analysis tools on the ExPASy Proteomics Server (http://expasy.org/) [37].

### Sequence alignment and phylogenetic analysis of PAT proteins

The PAT protein sequences were aligned using the ClustalX program with BLOSUM30 as the protein weight matrix. The MUSCLE program (version 3.52) was also used to perform multiple sequence alignments to confirm the ClustalX results (http://www.clustal.org/) [38]. Phylogenetic trees of the PAT protein sequences were constructed using the neighbour-joining (NJ) method of the MEGA5 program (http://www.megasoftware.net/) using the p-distance and complete deletion option parameters [39]. The reliability of the obtained trees was tested using a bootstrapping method with 1000 replicates. The images of the phylogenetic trees were drawn using MEGA5.

### The chromosomal location and structure of the PAT genes

The chromosomal locations and gene structures were retrieved from the genome data downloaded from the Phytozome database and PlantGDB database. The remaining genes were mapped to the chromosomes using MapDraw [40], and the gene structures of the PAT genes were generated with the GSDS (http://gsds.cbi.pku.edu.cn/) [41].

### Expression analyses of the PAT genes using GENEVESTIGATOR

The microarray expression data from various datasets were obtained using Genevestigator (https://www.genevestigator.com/gv/) [42] with the *Arabidopsis* (ATH1: 22k array), *Oryza sativa* (OS_51k: Rice Genome 51k array), *Zea mays* (ZM_84k: Nimblegen Maize 385k) and *Glycine max* (GM_60k: Soybean Genome Array) Gene Chip platforms. Then, the identified PAT-containing gene IDs were used as query sequences to perform searches in the Gene Chip platform of Genevestigator [42].

### Plant material and growth conditions

Qi319 (*Zea mays*) was used for this study. Unless stated otherwise, the seed germination and plant growth conditions were the same as those described by Ganal et al [43]. The primary root, pericarp, internode, adult leaf, silk, culm, seedling, endosperm, embryo and tassel were collected as described by Kong et al [44]. For the phytohormone treatments and abiotic stress assays, 5-day-old (*Zea mays*) wild type seedlings were transferred to liquid Murashige and Skoog (MS) medium [45] and supplemented with different treatments (or solvent control) for 6 h with gentle shaking. The aboveground seedlings were used in experiment. All the plant material was frozen in liquid N_2_ and stored at −80°C.

### RNA isolation and real-time PCR

Total RNA was isolated from the frozen tissue using the TRIzol reagent (Invitrogen) following the manufacturer's instructions. The RNA was further purified using a Fermentas RNAeasy mini kit. RNA (1 µg) was used as a template for first strand cDNA synthesis using the SuperScript First-Strand Synthesis system (Transgen). Real-time PCR was performed using gene-specific primers and the UltraSYBR Mixture (With Rox) (CWBio). The 18S ribosomal RNA genes were used as internal normalisation controls [46]. The fold changes in gene expression were calculated using the ΔCt values. To identify preferentially expressed genes, a student *t*-test was performed. A gene in a given tissue was defined as preferentially expressed only if the expression value of the gene in that tissue was more than 2-fold and had a *P* value less than 0.05 compared to other tissues. Under the phytohormone and abiotic treatments, the genes that were up or down-regulated more than 1.2-fold and with a *P* value less than 0.05 compared to the control were considered as differentially expression. The details of the primers used are listed in Table 1.

**Table 1:** The primers used for real-time PCR of *ZmPATs* genes in *Zea mays*.

Gene	Primers for real-time PCR (5′-3′)
*ZmPAT2*	Forward: CCTGGTGCAAAGCAAACA
	Reverse: CAGAGGACCTGGAGGATAGAG
*ZmPAT3*	Forward: CGACGACAGCGACCAAAT
	Reverse: GGCAAGTTCAGATCGGACATAG
*ZmPAT4*	Forward: TTCCTCATCATCGCACCA
	Reverse: CAAAGAAGCAGCGTCAAATC
*ZmPAT5*	Forward: GAATCCTTCGTCCTCAGCG
	Reverse: GATGGCGGGTTCTTCTCAA
*ZmPAT6*	Forward: TGCGGGTTACTCGCCTAT
	Reverse: ATGAGCACGGTGACAAAGAA
*ZmPAT8*	Forward: TGGCAATACGGCAAATCC
	Reverse: GCTATGAAGTGAGGCAATAAAGAG
*ZmPAT9*	Forward: TGGGCAGTCGATATTCTTCG
	Reverse: GCCTGTCCTCGCTTTCTCAC
*ZmPAT10*	Forward: ACCCATCCTCCTGAACCTG
	Reverse: GTCCCATTTACGATAACATCCTTT
*ZmPAT11*	Forward: AGCGGGAACCCTTCATTT
	Reverse: CAAGGTATCCAGCACAGTCTCA
*ZmPAT12*	Forward: CAGCAAGCCACTGAGGAA
	Reverse: AAGGGCGGTTGACATTAGA
*ZmPAT14*	Forward: GTGTTGAGCAGTTCGACCA
	Reverse: ATCCACGATTCTGAAGATGAG
*ZmPAT16*	Forward: GGAGGTGCCGCTGGTATA
	Reverse: CCTTCGCAACTAATGGACAG
*ZmPAT17*	Forward: GCCCTTTAGCATTGGCACT
	Reverse: CCATTTCCGATGTTCCTTGA
*ZmPAT18*	Forward: CTACCAGGCTTGGAAGGGAAAC
	Reverse: ATAGGAAGACAAGGTCCATGATCG
*ZmPAT19*	Forward: TTTGCGGAAGTGTTCTTTACC
	Reverse: TGTTCTGAAGTGCCGTTGG
*ZmPAT20*	Forward: GCCTCGCACAAAGGAAGT
	Reverse: CCAACAGAAGGCAAATACATAGAG
*ZmPAT21*	Forward: AAGAGTCCACCCATTCAGTAGAGG
	Reverse: GCACGTGTAACGAGCAGCTCTA
*ZmPAT24*	Forward: CACTGGGTTCCACTCTTATCTT
	Reverse: GGGTGCCTCATCTTCTCGT
*ZmPAT25*	Forward: TTGTTGAAGATGGGTTTGGA
	Reverse: AGCGGTCGCATGTAGAGC
*ZmPAT28*	Forward: AGATTCAGGAACCCGTATGAT
	Reverse: TGCGAAACCGCTCTTGTC
*ZmPAT29*	Forward: CATATTCGGACCTGACGCC
	Reverse: AAGATGCCGAGCAACGAAT
*ZmPAT30*	Forward: AGAAATGTTGCCACTATAAACCTC
	Reverse: TGTTCGAGAAGAATCGCTGC
*ZmPAT31*	Forward: TACTGGACGAGAAAGAAGGC
	Reverse: TGGGCACTGCTAATGGAG
*ZmPAT32*	Forward: TCCTCTGTGAATGTTGGTGGGT
	Reverse: CCCCTGACGCTTCATACCCA
*ZmPAT33*	Forward: GTTGCTCCCATTGCTCTATCT
	Reverse: TTCTGATCTTAATAATGAACACCC
*ZmPAT34*	Forward: CTGCGTTTCAGCATCCTGG
	Reverse: CTGCTCTAGCCGTTCAGTGTC
*ZmPAT35*	Forward: TCTCGGGCTTGTTTCCAC
	Reverse: ATGATGTAGTCTTGCCATTTGA
*ZmPAT36*	Forward: GATGGTTCCCCGCCTCTT
	Reverse: CCGTCACTCCGATGAACCT
*ZmPAT37*	Forward: TGACAAATCCAAGGGTTAGG
	Reverse: CAATGAAGTGAGGCAATAGAGA
*ZmPAT38*	Forward: CTTCGTGGCTGTGCTCGTC
	Reverse: TGCTACCGTGCAGTGAAATAC
*18S*	Forward: GATACCGTCCTAGTCTCAACC
	Reverse: GCCTTGCGACCATACTCC

## Results

### Identification of DHHC-CRD-containing PAT proteins in plants

To identify DHHC-CRD-containing PAT proteins in plants, we first collected the known 24 known PAT from *Arabidopsis* (Batistic, 2012) and analysed the domains of the PAT peptide sequences using PFAM search tool and SMART tool. Then, we used two different approaches to gather extensive information regarding this family. A total of 804 proteins were identified as potential members of the DHHC-CRD-containing PAT family within the 31 plant genomes that have been completely sequenced (Phytozome database: http://www.phytozome.net/). The presence of the DHHC-CRD domain in all of the protein sequences was confirmed using SMART and PFAM searches. We assigned each of these sequences an identity based on the gene identifier (Table S1).

**Figure 1:**
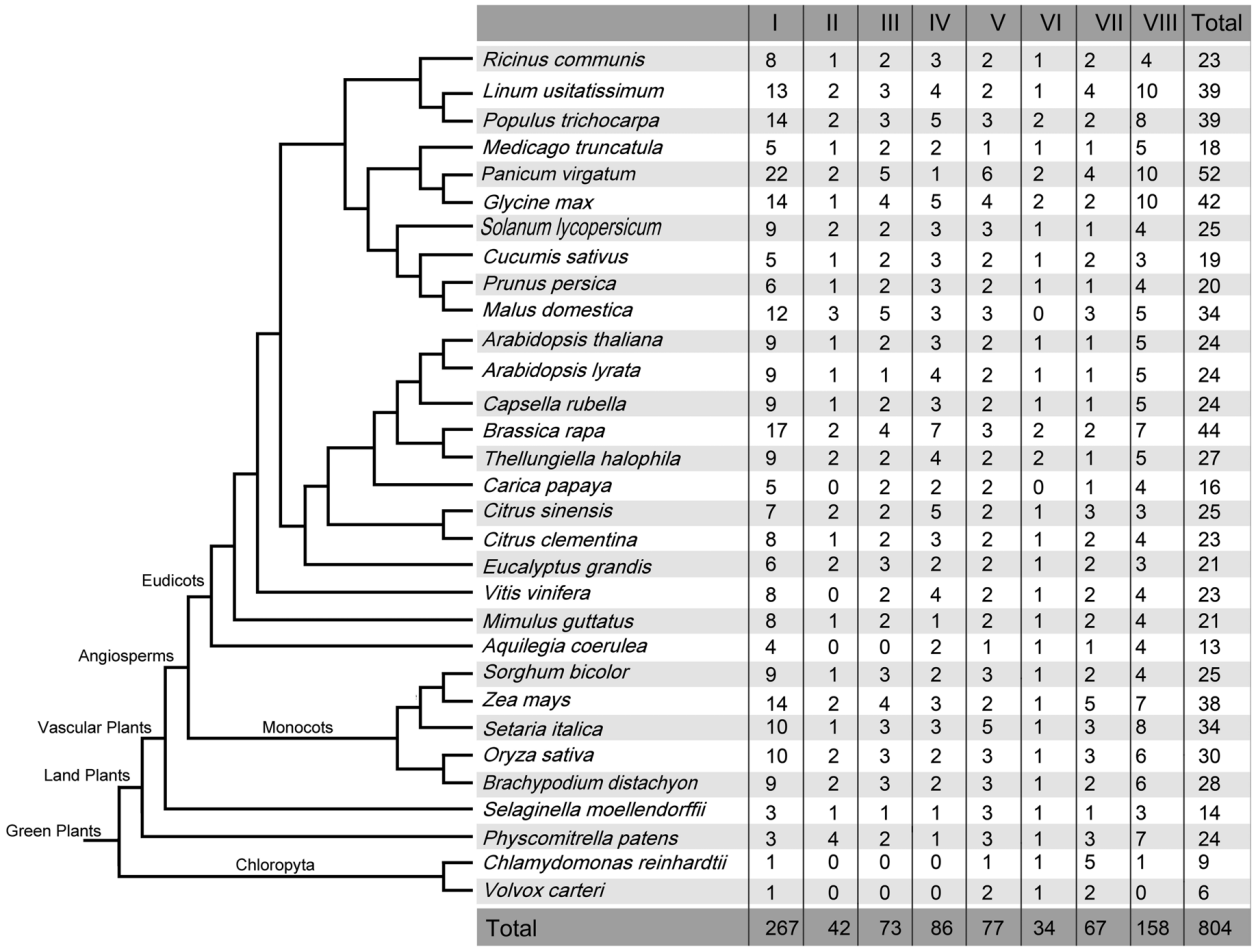
The phylogenetic relationships among the plants with completely sequenced genomes. The number in the table corresponds to the number of *PAT* genes in 8 subgroups and the total number of *PATs* in each species.

In this study, we observed that all 31 species contained PAT proteins and the number of PATs ranged from 6 (*Volvox carteri*) to 52 (*Panicum virgatum*) (Fig. 1). These results suggested that PATs are widely distributed in. Detailed information about the representative PAT proteins is included in Table S1. The protein length of the PATs ranged from 124 aa (BdiPAT26 and MdoPAT19) to 1024 aa (CrePAT3, MdoPAT6, MdoPAT24 and MdoPAT34), and the isoelectric point ranged from 4.82 (CsiPAT17) to 10.41 (SitPAT7) (Table S1).

### Phylogenetic relationships between the PATs family

**Figure 2:**
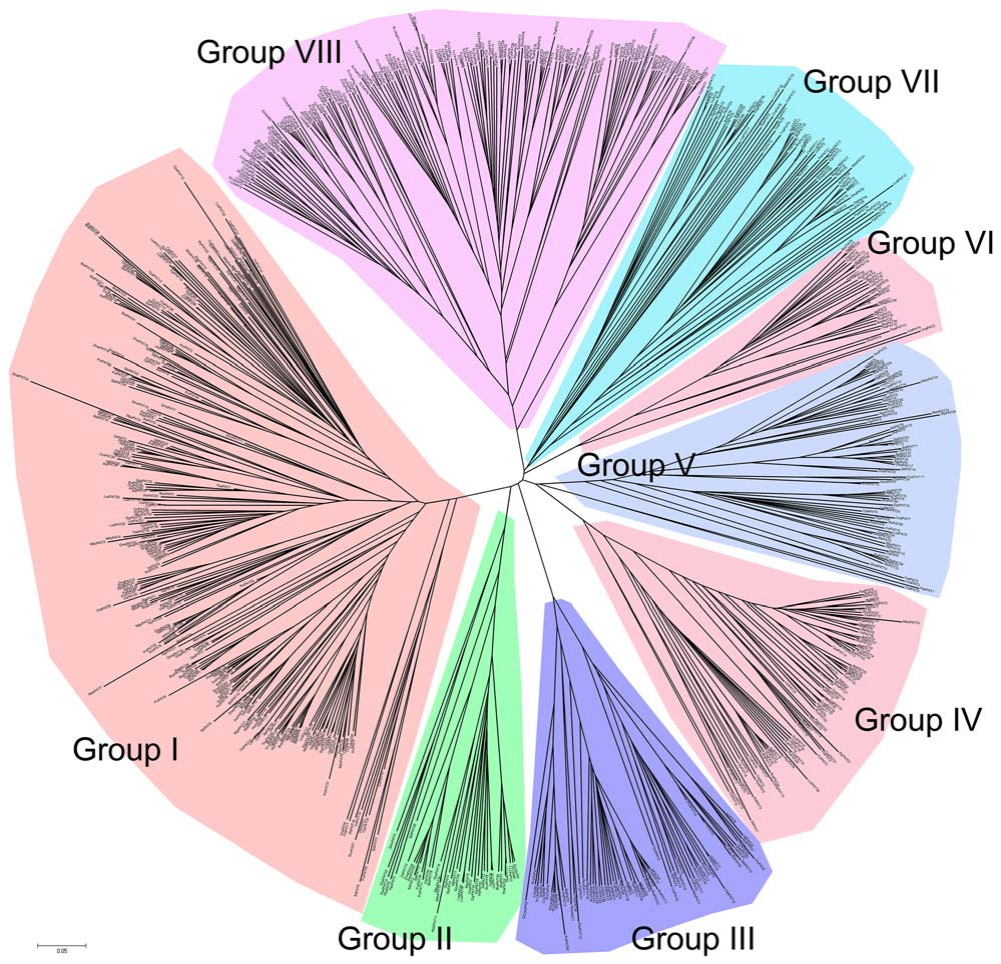
Phylogenetic relationships between the PATs in plants. The amino acid sequences of the plant PATs were aligned using MUSCLE, and the phylogenetic tree was constructed using the neighbour-joining method in the MEGA 5 software.

To clarify the phylogenetic relationship among the PATs proteins and to infer the evolutionary history of the protein family, the full-length protein sequences of the PATs in plants were used to construct a joint unrooted phylogenetic tree (Fig. 2), from which it could be observed that the proteins fell into 8 groups (group I to VIII). The number of PATs in each group was shown in Figure. 1. Statistically, 33.2% (267/804) of the PATs were in group I, which contained the highest number among the 8 groups. The lowest number of PATs was in group VI with 34 PATs, which was fewer than the total number of PATs in some species, such as *Panicum virgatum*, *Limum usitatissimum*, *Populustrichocarpa*, *Glycine max*, *Brassjca rapa* or *Zea mays*. In addition, we found that all the species from chloropyta to the green higher plants contained PATs from groups I, V and VII. In contrast, there were no PATs from group II, III or IV in chloropyta. These results suggested that the PATs in group II, III and IV may have originated after the divergence of ferns and chloropyta.

### Gene structure and chromosomal location of the PAT family in Arabidopsis, Oryza sative, Zea mays and Glycine max

**Figure 3:**
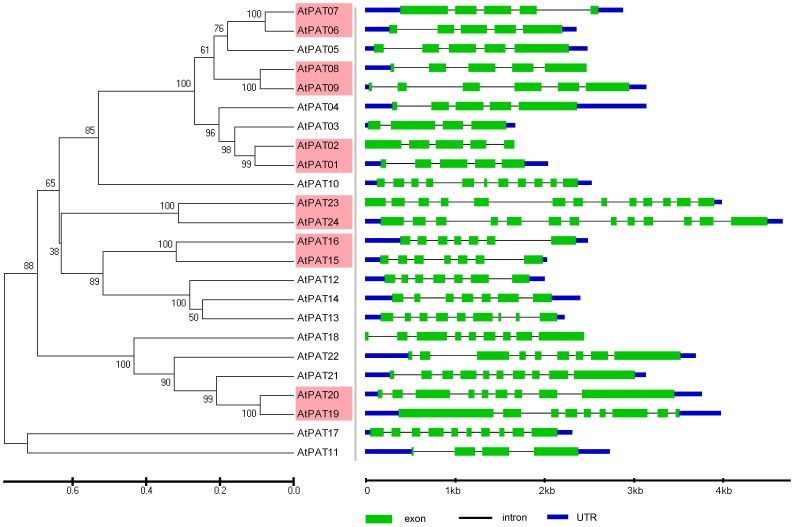
Evolutionary relationship and gene structure analysis of the AtPATs in *Arabidopsis*. The amino acid sequences of the AtPATs were aligned with Clustal X, and the phylogenetic tree was constructed using the neighbour-joining method in the MEGA 5.0 software program. Each node is represented by a number that indicates the bootstrap value. The scale bar represents 0.1 substitutions per sequence position (left). The right side illustrates the exon-intron organisation of the corresponding *AtPAT* gene. The exons and introns are represented by the green boxes and black lines, respectively. The scale bar represents 1 kb (right).

**Figure 4:**
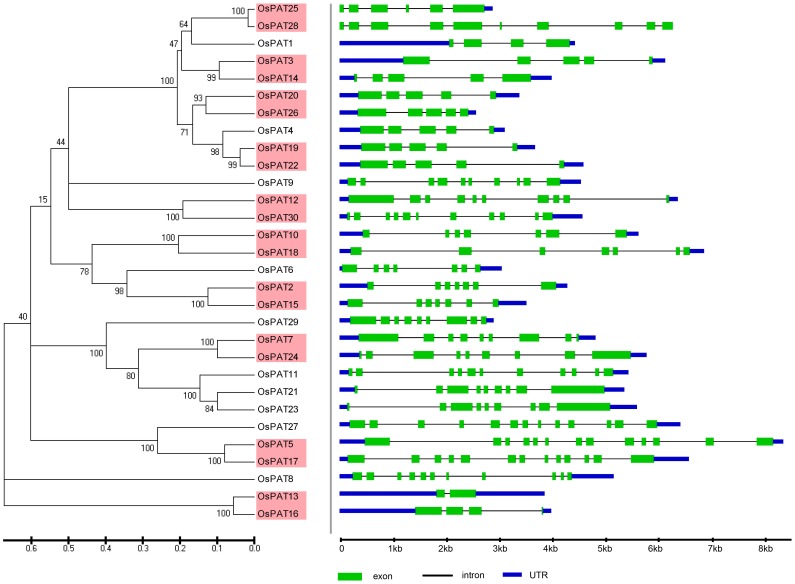
Evolutionary relationship and gene structure analysis of the OsPATs in *Oryza sative*. The amino acid sequences of the OsPATs were aligned with Clustal X, and the phylogenetic tree was constructed using the neighbour-joining method in the MEGA 5.0 software program. Each node is represented by a number that indicates the bootstrap value. The scale bar represents 0.1 substitutions per sequence position (left). The right side illustrates the exon-intron organisation of the corresponding *OsPATs* genes. The exons and introns are represented by the green boxes and black lines, respectively. The scale bar represents 1 kb (right).

**Figure 5:**
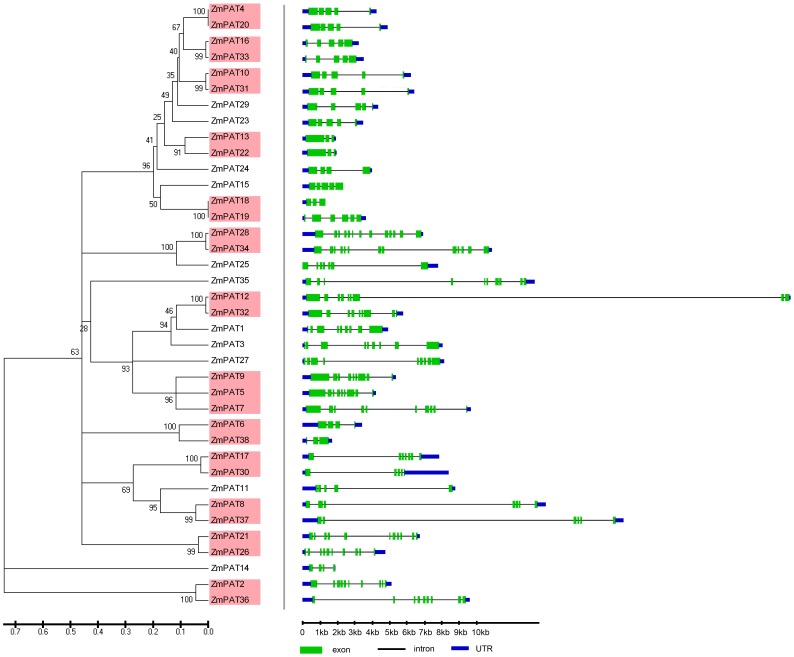
Evolutionary relationship and gene structure analysis of the ZmPATs in *Zea mays*. The amino acid sequences of ZmPATs were aligned with Clustal X, and the phylogenetic tree was constructed using the neighbour-joining method in the MEGA 5.0 software program. Each node is represented by a number that indicates the bootstrap value. The scale bar represents 0.1 substitutions per sequence position (left). The right side illustrates the exon-intron organisation of the corresponding *ZmPATs* genes. The exons and introns are represented by the green boxes and black lines, respectively. The scale bar represents 1 kb (right).

**Figure 6:**
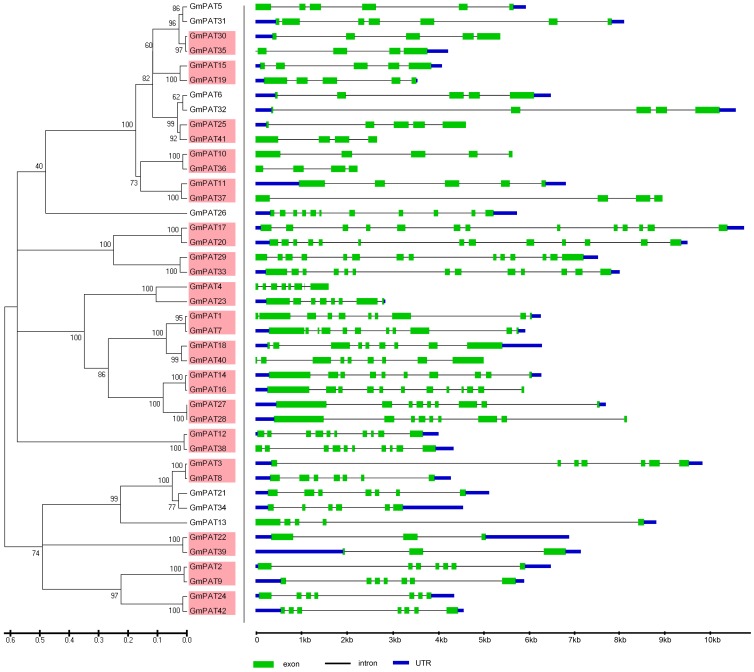
Evolutionary relationship and gene structure analysis of the GmPATs in *Glycine max*. The amino acid sequences of GmPATs were aligned with Clustal X, and the phylogenetic tree was constructed using the neighbour-joining method in the MEGA 5.0 software program. Each node is represented by a number that indicates the bootstrap value. The scale bar represents 0.1 substitutions per sequence position (left). The right side illustrates the exon-intron organisation of the corresponding *GmPATs* genes. The exons and introns are represented by the green boxes and black lines, respectively. The scale bar represents 1 kb (right).

The phylogenetic relationships of the PATs in *Arabidopsis, Oryza sative, Zea mays* and *Glycine max* were examined by aligning their amino acid sequences and implementing the neighbour-joining method in MEGA 5.0. Then, a more extensive bioinformatic analysis of the PAT gene family was subsequently performed. We compared the genomic sequences of all of the genes with the sequences from the Gene Structure Display Server (GSDS, http://gsds.cbi.pku.edu.cn/.) to generate schematic diagrams of the gene structure. An analysis of the structure of the *PATs* genes showed that the mosaic structure was different from each member of the DHHC-CRD-containing *PATs* gene family in *Arabidopsis, Oryza sative, Zea mays* and *Glycine max*, respectively. Not only were the positions and lengths of the introns not conserved, but the number of introns was also highly variable (Fig. 3–6). The number of introns ranged from 3 to 12 in *Arabidopsis*, from 2 to 12 in *Zea mays*, from 1 to 12 in *Oryza sative* and from 2 to 13 in *Glycine max*. Among the four plants, we found that most of the PATs contained 4 introns, and the number of PATs containing 4 introns was 7 in *Arabidopsis*, 11 in *Zea mays*, 7 in *Oryza sative* and 10 in *Glycine max*. In addition, we found that the length of most of the PATs genes in *Arabidopsis* was shorter than that in *Oryza sative*, *Zea may* or *Glycine max*, which could account for the short introns.

**Figure 7:**
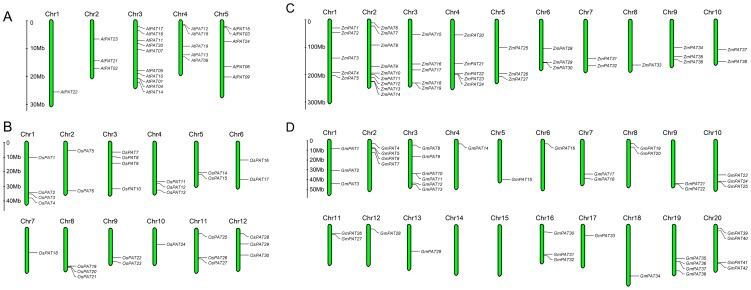
Chromosomal locations of *PATs* genes in *Arabidopsis*, *Oryza sative*, *Zea mays* and *Glycine max*. (A)Chromosomal locations of At*PATs* genes in *Arabidopsis*. (B)Chromosomal locations of OsPATs genes in *Oryza sative*. (C)Chromosomal locations of ZmPATs genes in *Zea mays*. (D)Chromosomal locations of GmPATs genes in *Glycine max*.

To determine the genomic distribution of the *PATs* genes in *Arabidopsis, Oryza sative, Zea mays* and *Glycine max*, we used their DNA sequences to search the Phytozome database and the PlantGDB database. The position of each gene is shown in Fig. 7. The chromosomal location analysis showed that the *AtPATs*, *OsPAT* and *ZmPATs* were distributed across all chromosomes with different densities in *Arabidopsis* (Fig. 7A), *Oryza sative* (Fig. 7B) and *Zea mays* (Fig. 7C), respectively. However, 42 *GmPATs* genes were found to be distributed across 18 chromosomes, and there was no *GmPATs* located on chromosomes 14 or 15 (Fig. 7D).

### Expression profiling of PATs genes in Arabidopsis, Oryza sative, Zea mays and Glycine max

To investigate the expression profiling of the *PATs* gene in plants, we used bioinformatics methods to gather extensive microarray information regarding this family in the model plant, *Arabidopsis* and in important crops (*Oryza sative*, *Zea mays* and *Glycine max*). The plant tissues and developmental stages selected for microarray analysis span the entire life cycle. The deep and light colour shading represents the relatively high or low expression levels of the *PATs* genes in the different tissues, respectively.

**Figure 8:**
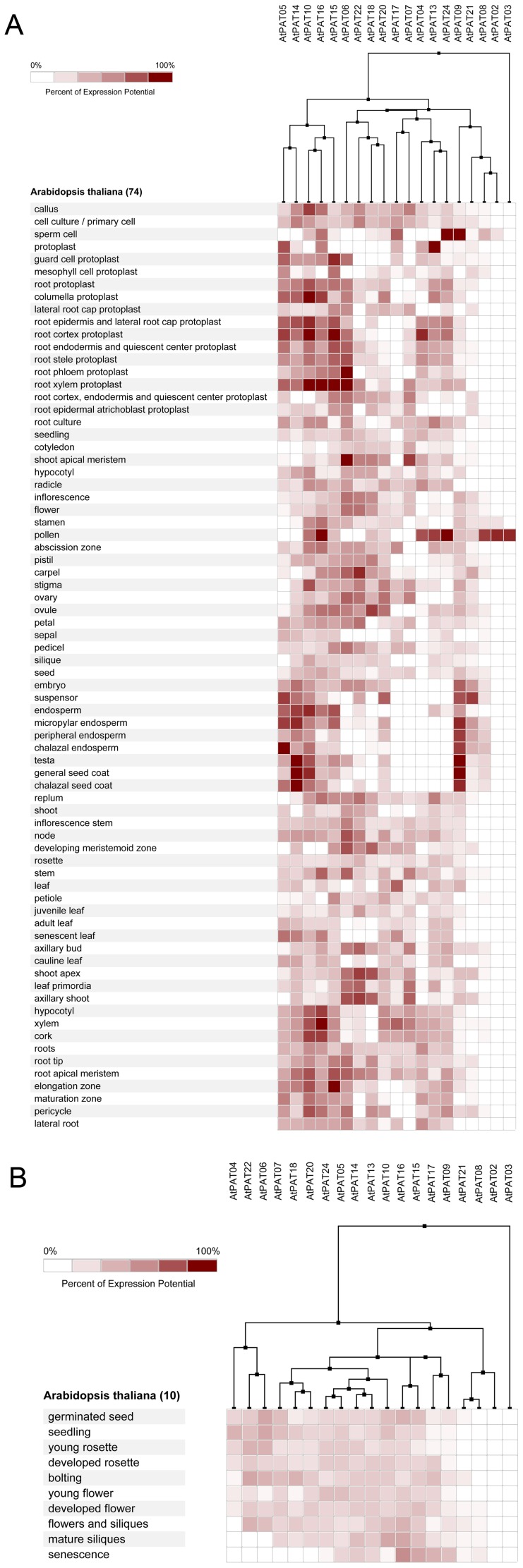
Expression analysis of *AtPATs* genes in *Arabidopsis*. The heatmap was prepared using the Genevestigator tool, and the microarray expression data were from the results of many chips available on the web (https://www.genevestigator.com/gv/). The dark and light colour shadings represent relatively high or low expression levels, respectively. (A)Expression analysis of *AtPATs* genes in different tissues. (B)Expression analysis of *AtPATs* genes in different life stages

**Figure 9:**
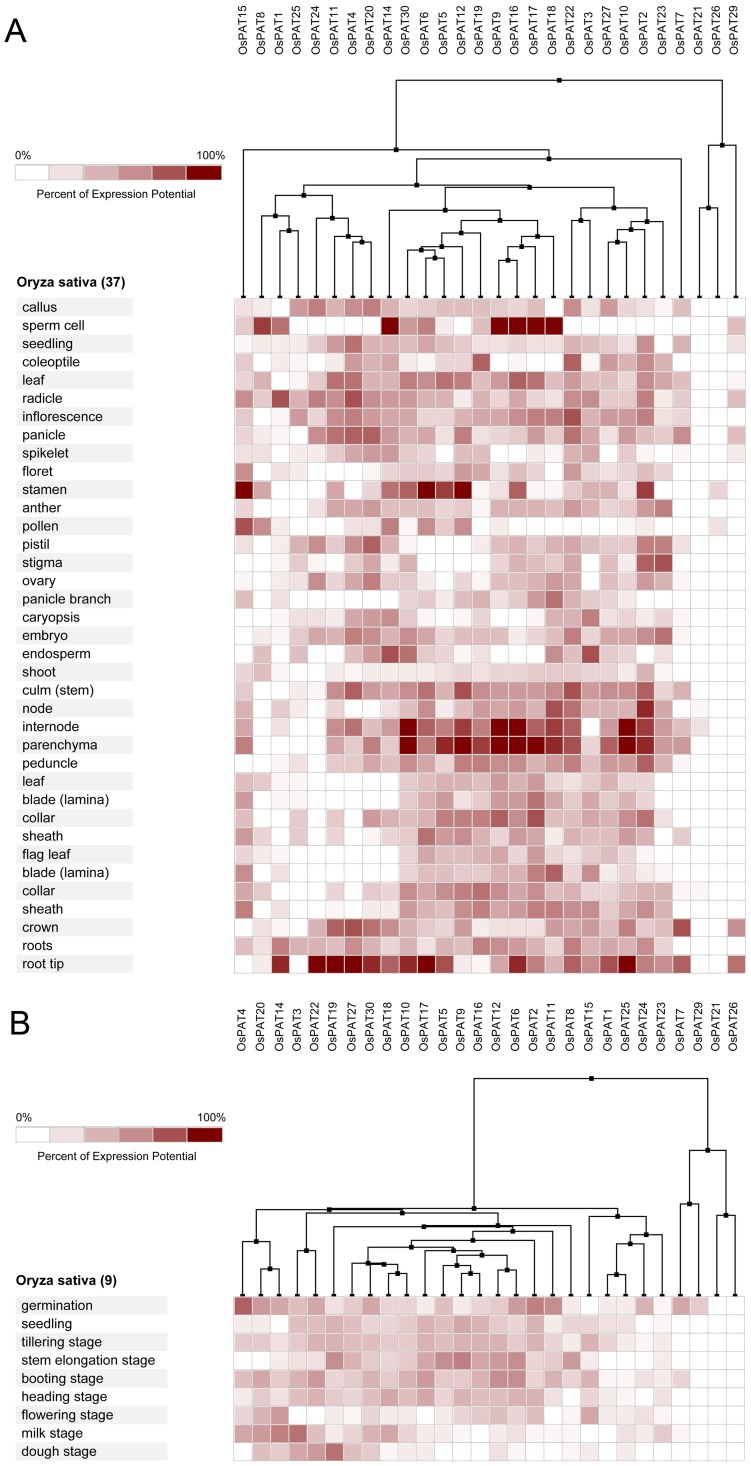
Expression analysis of *OsPATs* genes in *Oryza sative*. The heatmap was prepared using the Genevestigator tool, and the microarray expression data were from the results of many chips available on the web (https://www.genevestigator.com/gv/). The dark and light colour shadings represent relatively high or low expression levels, respectively. (A)Expression analysis of *OsPATs* genes in different tissues. (B)Expression analysis of *OsPATs* genes in different life stages

**Figure 10:**
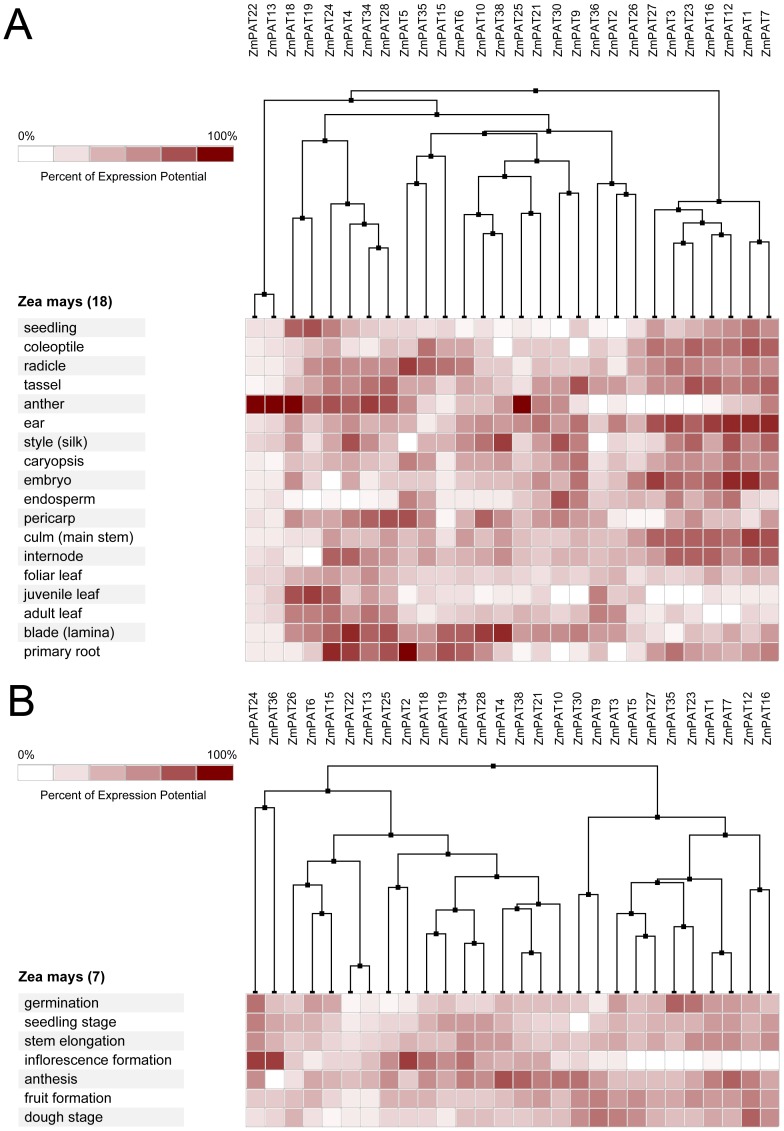
Expression analysis of *ZmPATs* genes in *Zea mays*. The heatmap was prepared using the Genevestigator tool, and the microarray expression data were from the results of many chips available on the web (https://www.genevestigator.com/gv/). The dark and light colour shadings represent relatively high or low expression levels, respectively. (A)Expression analysis of *ZmPATs* genes in different tissues. (B)Expression analysis of *ZmPATs* genes in different life stages

**Figure 11:**
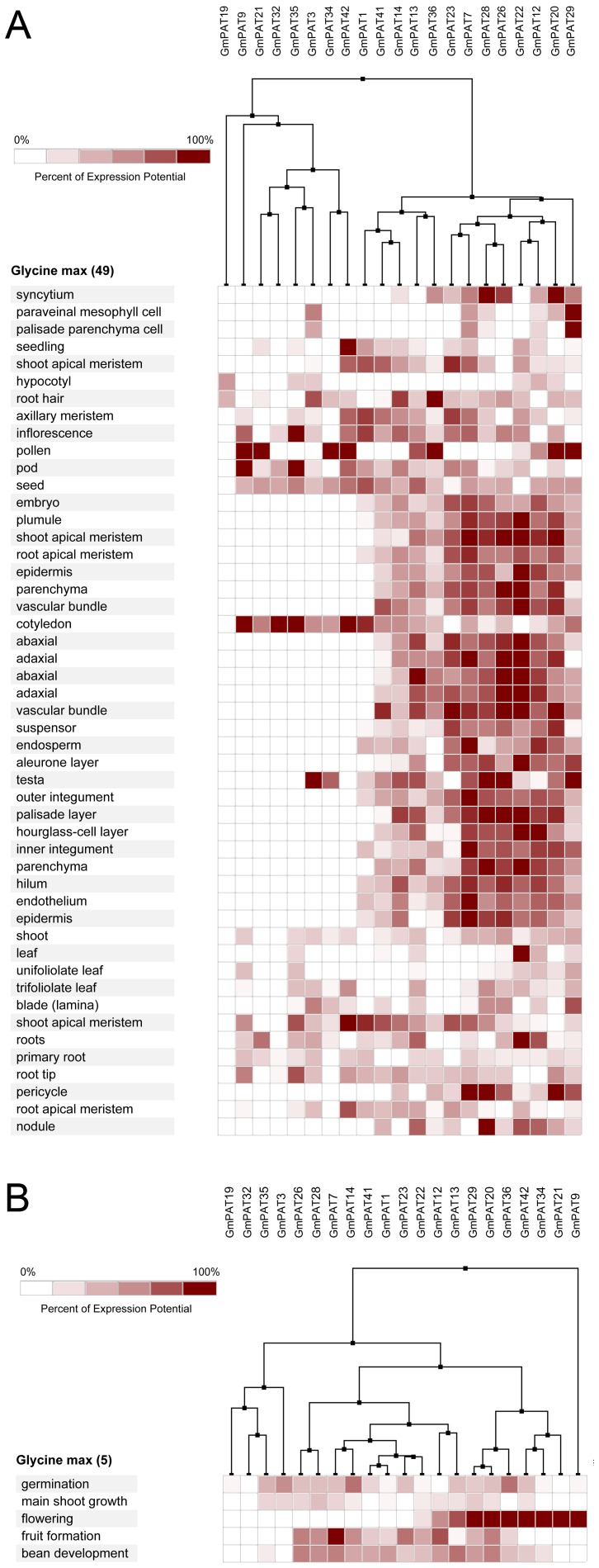
Expression analysis of *GmPATs* genes in *Glycine max*. The heatmap was prepared using the Genevestigator tool, and the microarray expression data were from the results of many chips available on the web (https://www.genevestigator.com/gv/). The dark and light colour shadings represent relatively high or low expression levels, respectively. (A)Expression analysis of *GmPATs* genes in different tissues (B)Expression analysis of *GmPATs* genes in different life stages

In *Arabidopsis*, 19 of the 24 *AtPATs* genes could be identified using microarray. Most *AtPATs* showed a broad expression pattern at different developmental stages and tissues analysed, except *AtPAT2* and *AtPAT3*, which only exhibited high expression in the stamen. In addition, *AtPAT8* and *AtPAT21* exhibited very low expression in the developmental stages (Fig. 8). In *Oryza sative*, 28 of the 30 *OsPATs* genes could be identified. The results showed that *OsPAT21* and *OsPAT26* were only expressed in the internode and stamen, respectively. However, the other 26 *OsPATs* showed transcript abundance in more than one tissue. Interestingly, we found that *OsPAT29* was specifically expressed during germination and was expressed in many tissues (Fig. 9). This finding accounted for the different amounts of RNA in the tissues in different developmental stages. In *Zea mays*, 28 of 38 *ZmPATs* were analysed. The results showed that all 28 *ZmPATs* exhibited extensive expression in the developmental stages and tissues analysed. In addition, *ZmPAT13* and *ZmPAT22* showed similar expression patterns and exhibited high expression in the anther (Fig. 10). In *Glycine max*, only half of the *GmPATs* were analysed. Among the 21 *GmPATs*, 7 genes (*GmPAT9, 20, 21, 29, 32, 34, 42*) were specifically expressed in the flowering stage, while 11 genes (*GmPAT1, 3, 7, 14, 19, 23, 26, 28, 32, 35, 41*) were not expressed in the flowering stage. In addition, *GmPAT19* and *GmPAT32* were abundantly transcribed in they hypocotyl and cotyledon, respectively (Fig. 11).

**Figure 12:**
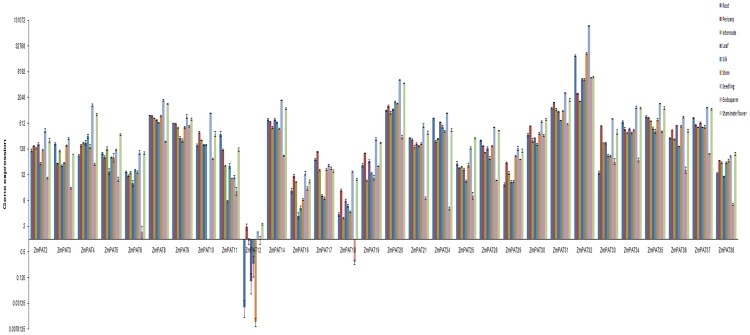
Quantitative RT-PCR to measure the expression patterns of 30 *ZmPATs* genes in *Zea mays*. Error bars indicate standard deviations (n = 3). 1, primary root; 2, pericarp; 3, internode; 4, adult leaf; 5, silk; 6, culm; 7, seedling; 8, endosperm; 9, embryo; 10, tassel.

With the aim of revealing the characteristics of *ZmPATs* expression, an analysis of the preferential expression of 30 *ZmPATs* was performed using real-time PCR with gene-specific primers in *Zea mays*. Among the 30 genes, *ZmPAT32* had the highest expression level, and *ZmPAT12* had the lowest expression level in the tissues tested. In addition, we found that most of the genes (19 *ZmPATs*) had higher expression signals in the seedlings and flowers than in the other tissues tested, and many genes (17 *ZmPATs*) showed the lowest expression levels in the endosperm (Fig. 12). These results demonstrated that the expression of the *ZmPATs* was extensive in different tissues and exhibited a preferential expression profile.

### Responses of *ZmPATs* to phytohormones and abiotic stress in *Zea mays*

**Figure 13:**
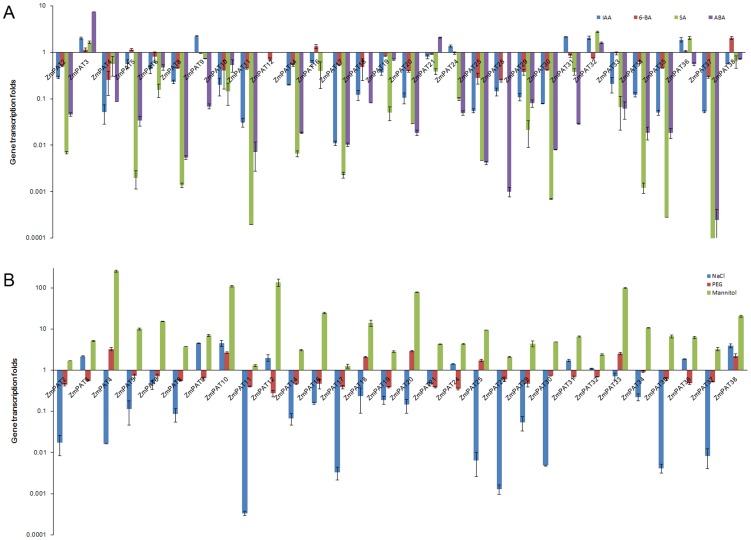
The expression profiles of some *ZmPATs* genes response to phytohormones and mimic abiotic stress. (A)The expression profiles of some *ZmPATs* genes are responsive to phytohormones. (B)The expression profiles of some *ZmPATs* genes mimic the response to abiotic stress. 5-day-old (*Zea mays*) wild type seedlings were transferred to liquid MS media supplemented with 5 µM 6-BA, 5 µM IAA, 100 µM SA, 100 µM ABA, 100 mM NaCl, 300 mM mannitol or 15% PEG 6000 (or solvent control) for 6 h with gentle shaking. Representative experiments are shown, and the experiments were performed three times. Each bar represents a mean±SEM (n = 3).

Phytohormones play a critical role in plant growth and development. To investigate the potential function of the *PATs* genes in plants, we surveyed the responses of the *ZmPATs* to phytohormones. The analysis was performed using the total RNA from the leaves of seedlings treated with IAA (indole-3-acetic acid), 6-BA (6-benzylaminopurine), SA (salicylic acid) and ABA (abscisic acid). The real-time PCR results showed that 14 *ZmPATs* were down regulated by all of the phytohormones tested. In additional, 6 *ZmPATs* (*ZmPAT3, 9, 24, 31, 32 and 36*) were up-regulated by IAA, 2 *ZmPATs* were up-regulated by 6-BA (*ZmPAT16 and 38*) and 2 *ZmPATs* (*ZmPAT3 and 32*) were up-regulated by both SA and ABA (Fig. 13A). Therefore, the results demonstrated that phytohormones affected the expression of *ZmPATs* and suggested that these proteins may play roles in plant growth and development.

Previous reports have revealed that SA and ABA mediate the plant's response to stress by rapid accumulation, thus facilitating plant survival. Nearly all of the *ZmPAT* genes tested were up or down regulated by SA and ABA. The results suggested that the *ZmPATs* may be involved in the signalling pathways that are triggered by abiotic stresses. To determine the responses of these genes to different abiotic stresses, an experimental analysis was performed using the total RNA from the leaves of seedlings treated with NaCl, PEG and mannitol. The results showed that *ZmPAT10* and *ZmPAT38* were induced by all the treatments, and all the *ZmPAT* genes were induced by mannitol, with the exception of *ZmPAT17*. In addition, the expression levels of 57% of the *ZmPATs* (17/30) were induced or reduced by both NaCl and PEG. Six genes (*ZmPAT3, 9, 12, 24, 31, 36*) were up-regulated by NaCl and down-regulated by PEG. On the contrary, 5 genes (*ZmPAT4, 18, 20, 25, 33*) were down-regulated by NaCl and up-regulated by PEG (Fig. 13B). These results suggest that the functions of the *ZmPAT* genes may be involved in the responses to stresses in *Zea mays.*

## Discussion

DHHC-CRD-containing proteins mediate the S-acylation of proteins [1], [19]. However, the studies of plant PATs are still very limited. To identify additional plant PATs and to infer their biological functions and investigate the evolutionary history of this protein family, we identified 804 putative PATs in 31 plants with completely sequenced genomes. Notably, the DHHC-CRD PATs were found in both the vascular and non-vascular plants as well as in green algae. This result demonstrated the important roles and relativly conservative evolution of PATs in plant life. In addition, the number of PATs was significantly different, ranging from 6 (*Volvox carteri*) to 52 (*Panicum virgatum*). Generally, the numbers of PATs is fewer in low plant than which in green higher plant. In average, there are 27.2 PATs in land plants, while only 7.5 PATs in chloropyta. This may be due to substrate specificity and the redundancy of genes that serve important functions in plant evolution processing. The first systematic analysis of how individual DHHC proteins affect cellular palmitoylation was performed in *S. cerevisiae* [16]. The proteomic analysis of the substrate palmitoylation profiles in DHHC-deficient yeast strains provided clear evidence for substrate selectivity: most Akr1 target are soluble proteins that are exclusively palmitoylated; Erf2 substrates tend to be modified by myristoyl or prenyl groups; and Swf1 has a preference for cysteine residues in proximity to TMDs [47], [48]. Moreover, an analysis of DHHC-substrate interactions based on co-expression was performed in mammals, and the results suggested that some DHHC palmitoylated a broad range of substrates [1], whereas others were more selective, such as DHHC4, 5, 9 and 12 [49]-[51]. Therefore, there are numerous PATs in some higher plants that may be more selective.

To clarify the phylogenetic relationships among the PAT proteins and infer the evolutionary history of the protein family, a phylogenetic tree of PATs in plants was constructed. It could be observed that the proteins fell into 8 groups (group I to VIII). The sequence conservation among the groups of PATs is exceptional low, which is consistent with AtPATs[21]. This indicates an overall early and differential evolution of the groups. In addition, we found that there were no PATs in groups II, IIIand IV in chloropyta. The reason for this phenomenon may be the complex localisation of PATs in the green higher plants. The tissue and cellular localisation of the PATs is more complex in green higher plants than algae. In Arabidopsis, the expression patterns of 20 *PAT* genes were studied. Nine *AtPATs* (*AtPAT1, 3, 5, 7, 9, 10, 11, 22, 24*) have high expression in flowers, 5 *AtPATs* (*AtPAT12, 14, 16, 17, 23*) are highly expressed in seedlings and 8 *AtPATs* (*AtPAT4, 5, 6, 8, 13, 14, 15, 22*) exhibit high expression in siliques. In addition, the cellar localisation of all 24 AtPATs was studied. Most of these proteins are targeted to membrane structures, such as the endoplasmic reticulum, the Golgi, endosomes and the vacuolar membrane [7]. This complex targeting pattern of PATs demonstrates that S-acylation, which is different in plants than in yeast and mammals, can occur at different cellular membranes within the plant cell.

Knowing the expression profiles of some *PAT* genes may provide clues as to the biological function of PATs. Through the analysis of the expression profiles in *Arabidopsis*, *Oryza sative*, *Zea mays* and *Glycine max*, we found that *PAT* gene transcripts accumulated during different developmental stages and in different tissues (Fig. 5, 6). The variability in the expression patterns of the genes in the same family indicates that their roles might not be redundant and that these genes, which are preferentially expressed in specific tissues, may require further investigation to fully understand their functions. In addition, crops and the model plant *Arabidopsis* were used in this expression analysis. Therefore, the results from this study will be useful for further studying crop production.

S-acylated proteins play a wide variety of roles in plants and affect Ca^2+^ signalling [52], [53], K^+^ movement [3], stress signalling [26], small and heteotrimeric G-protein membrane association an partitioning [54], tubulin function and pathogenesis [3], [55]. Therefore, knowing the response of the *PAT* genes to phytohormones and stress treatments may provide clues to their function. Among the 31 plants that have been completely sequenced, we used the model crop *Zea mays* as an example. Through our transcriptional analysis of the *ZmPATs*, we found that the expression of all the examined genes was affected by exposure to phytohormones. This result suggests that the PATs likely play roles in plant growth and development. In addition, nearly all the *ZmPAT* genes tested were up or down regulated by SA and ABA, which mediates the plant's response to stress, specifically abiotic stress. Accordingly, all the *ZmPATs* were affected by NaCl, PEG or mannitol, and many genes were affected by all three treatments, such as *ZmPAT4, 10, 20* and *38*. These results inferred that the functions of the *PATs* genes may be involved in responses to stresses in plants.

In conclusion, the preferential expression of the *PATs* genes in specified tissues and their response to phytohormones and stress treatments provide clues to the roles of these genes in signalling, growth and development. The systematic sequence analysis and expression profiles of the *PATs* genes will serve as a useful reference for more detailed functional analyses and will be helpful in the selection of appropriate candidate genes for further studies and genetic engineering.

## Supporting Information

Table S1 **Putative DHHC-Cysteine-Rich Domain S-Acyltransferase in Plants.**
(XLS)
